# Role of Physicochemical Properties in Nanoparticle Toxicity

**DOI:** 10.3390/nano5031351

**Published:** 2015-08-19

**Authors:** Seung Won Shin, In Hyun Song, Soong Ho Um

**Affiliations:** 1 School of Chemical Engineering, Sungkyunkwan University, Suwon, Gyeonggi-do 440-746, Korea; E-Mails: seung3885@skku.edu (S.W.S.); songinhyun@skku.edu (I.H.S.); 2 SKKU Advanced Institute of Nanotechnology (SAINT), Sungkyunkwan University, Suwon, Gyeonggi-do 440-746, Korea

**Keywords:** nanotoxicology, nanomaterials, physiological properties

## Abstract

With the recent rapid growth of technological comprehension in nanoscience, researchers have aimed to adapt this knowledge to various research fields within engineering and applied science. Dramatic advances in nanomaterials marked a new epoch in biomedical engineering with the expectation that they would have huge contributions to healthcare. However, several questions regarding their safety and toxicity have arisen due to numerous novel properties. Here, recent studies of nanomaterial toxicology will be reviewed from several physiochemical perspectives. A variety of physiochemical properties such as size distribution, electrostatics, surface area, general morphology and aggregation may significantly affect physiological interactions between nanomaterials and target biological areas. Accordingly, it is very important to finely tune these properties in order to safely fulfill a bio-user’s purpose.

## 1. Introduction

**Figure 1:**
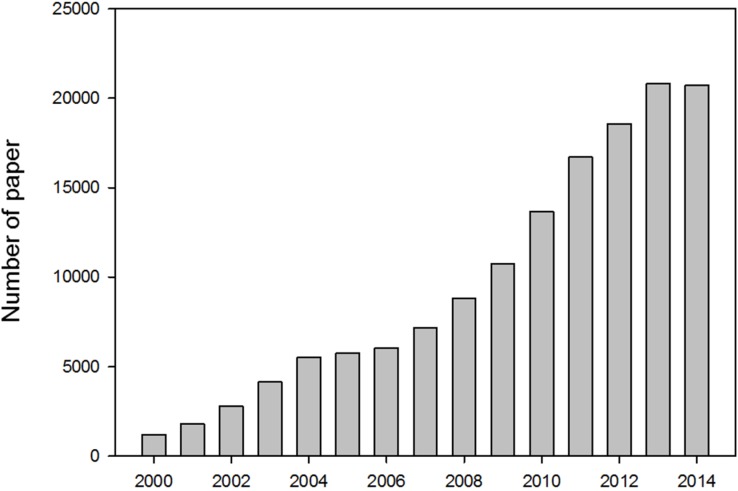
The number of nanoscience papers indexed in Scopus between 2000 and 2014. Source: “Nanoparticle”, data from Scopus.

Since basic concepts of nanotechnology were introduced in the mid-twentieth century by Richard Feynman, Norio Taniguchi and Eric Drexler, a large body of literature on nanomaterials has accumulated and has expanded significantly in the twenty first century (Figure 1 and Table 1). Furthermore, the rapid development of methods and tools for nanomaterial/colloid characterization has resulted in substantial advances in materials (*i.e.*, fullerene and carbon nanotubes) [1]. Inspired by these trends, many scientists now favor nanomaterials with new characteristics over outdated bulky materials. As of the early 2000s, these newer materials have begun to deepen their impact on our daily lives in applications such as clothing, cosmetics, furniture, and even foods.

**Table 1:** Detail information about the graph was described in the table. Highly cited authors, country, and subject area are used as specific subsection of papers. Source: “Nanoparticle”, data from Scopus.

Author	Papers	Country	Papers	Subject area	Papers
Couvreur, P.	250	United States	39,702	Chemistry	62,277
Mirkin, C.A.	244	China	31,406	Materials Science	59,895
Rotello, V.M.	227	India	10,590	Physics and Astronomy	40,126
Muller, R.H.	199	Germany	10,180	Chemical Engineering	40,108
Kreuter, J.	186	Japan	9,951	Engineering	36,940
Weissleder, R.	185	South Korea	9,118	Biochemistry	30,209
				Genetics and Molecular Biology	
Yuan, R.	170	United Kingdom	6,809	Medicine	21,275
Xia, Y.	149	France	6,484	Pharmacology	18,456
				Toxicology and Pharmaceutics	
Lanza, G.M.	148	Italy	4,457	Environmental Science	8,709
Wickline, S.A.	146	Spain	4,306	Others	21,726

The nanomaterials market has exploded. For example, titanium dioxide (TiO_2_) nanoparticles, which are a major ingredient in sunblock, absorb ultraviolet (UV) light and efficiently protect skin from harmful UV light exposure. They are also used in many cosmetics. In addition, silica dioxide (SiO_2_) is often used as a food additive to decrease viscosity and regulate acidity. Owing to their powerful anti-microbial and light-weight characteristics, silver (Ag) nanoparticles and carbon nanotubes (CNTs) are used extensively in a variety of cleansers and in sporting equipment. However, its safety is continuously debated among many scientists and clinicians.

The cytotoxicity of nanoparticles is induced by several factors. Some cases of nanomaterials inducing cytotoxicity are because of the substance itself, and some nanoparticles show toxicity without clear mechanism [2,3]. Some nanoparticles of a particular substance are thought to pose greater risks of toxicity than larger-sized particles of the same substance [4,5,6]. Above all, the distribution of particles within the body and the accumulation of a specific type of particle in a particular part of the body, which is dependent on the particle’s size and surface characteristic, are considered critical issues [7]. Also, when the nanoparticles accumulate in body system without proper excretion, it can cause continuous toxicity. The main distribution sites and target organs for nanoparticles are unknown; however it appears that the liver and spleen may be target organs [8,9]. If nanoparticles are ingested, inhaled or absorbed through the skin, they can induce the formation of reactive oxygen species (ROS) including free radicals [10]. ROS produces oxidative stress, inflammation, and consequent damage to various biological materials such as protein, DNA, *etc.* Besides ROS production, other factors influencing toxicity include size, morphology, agglomeration statue, shape, chemical composition, surface structure, surface charge, aggregation and solubility [11]. As a result of their small size, nanoparticles can cross tissue junctions and even cellular membranes where they induce structural damage to the mitochondria [12,13] or invade the nucleus where they cause serious DNA mutations [14] leading to cell death [15]. The factors mentioned above can be categorized under the five characteristics of nanoparticle, which are: size; surface area; electrostatic statue of surface; morphology; and agglomeration status.

Researchers have made a substantial effort to minimize unwanted interactions between nanomaterials and biological tissues. Researchers have investigated surface coatings and other modifications to increase the safety of nanoparticles in the body. However, these surface coatings are protective for only a short time because they are destroyed over a period of one to four hours by environmental interactions such as air exposure or ultraviolet irradiation [16,17]. To overcome this shortage, several trials have been conducted and have shown a link between long term coating of nanoparticles and a significant reduction of cytotoxicity [18].

**Figure 2:**
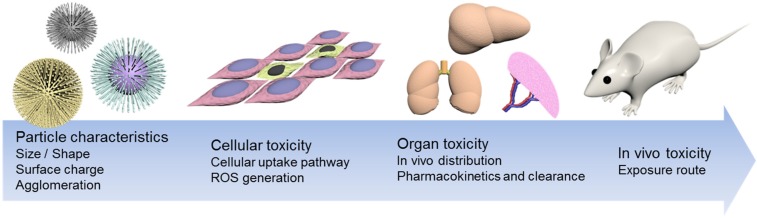
Schematic drawing of nanoparticle induced cytotoxicity. Intrinsic features of nanoparticles, such as size, surface charge, agglomeration, can significantly affect cytotoxicity. Such cytotoxicity can be affected at the levels of the cell, organ and even *in vivo* systems.

Some say that toxicological data for nanoparticles is lacking because of the short history of nanotechnology in healthcare. However, others insist that nanomaterials are safe for healthcare use [19,20]. To settle the debate, it is necessary to clarify the physicochemical properties of nanoparticles related to toxicity. In principle, nanomaterials distinguishable characteristics are mainly assumed to originate from either their surface area or mass quantity, and their lifespan is based on biological cellular interactions. Some of them are unstable at the surface, showing unusual communication with their biological neighbors. Many recent studies have shown that this instability could be minimized by modulating some physicochemical properties. In this short review, recent trends regarding the role of nanomaterials’ physicochemical properties, in terms of *in vitro* and *in vivo* toxicological results, will be discussed (Figure 2).

## 2. Size

Cytotoxicity is induced by nanomaterials results from the interaction between the nanomaterial surface and cellular components. As the diameter decreases, the surface area of the particle increases exponentially. Thus, even when particles have the same composition, they can have significantly different levels of cytotoxicity depending on both particle size and surface reactivity. Additionally, particle size induces significant differences in the cellular delivery mechanism and distribution *in vivo*. In this regard, not only are chemical properties and size-dependent cytotoxicity important in assessing a nanomaterial’s cytotoxicity, but also is the amount of size-dependent cytotoxicity.

### 2.1. Size-Dependent Absorption

To generate cytotoxicity and inflammatory response in animal models, it is essential that the nanoparticles should migrate across the epithelial barrier. In this respect, the size of the nanoparticles plays a key role in cytotoxicity [21,22]. In the case of nanoparticle inhalation, nanoparticles penetrate deeply into the lung parenchyma. Different sized nanoparticles show specific distribution patterns in the respiratory tract. Nanoparticle distribution is also affected by the Stokes number and Reynolds number. Initially, particles are well distributed in the gas phase, but after inhalation they translocate into the liquid phase in respiratory fluids [23,24].

Recently, Braakhuis *et al*. assessed cytotoxicity induced by inhaled silver nanoparticles of different size [25]. They prepared 18, 34, 60, and 160 nm silver nanoparticles and exposed rats to different concentrations of the particles. After exposure, the rats were sacrificed and the amount of silver nanoparticles in their lungs was measured. They found that silver nanoparticles in sizes of 18 and 34 nm induced lactate dehydrogenase (LDH) expression, which is a marker of cell damage, in a dose-dependent manner after 24 h. Meanwhile, there was no dose-dependent cell damage when 60 and 160 nm nanoparticles were used. Although there were more 60 and 160 nm nanoparticles measured in the lungs overall, more of the 18 and 34 nm nanoparticles were found in the alveoli. The authors indicated that the increased surface area of the nano-scaled particles was the most likely factor contributing to the toxicology of the silver nanoparticles.

### 2.2. Size-Dependent in Vivo Pharmacokinetics and Clearance

The distribution of a drug or nanoparticles *in vivo*, or pharmacokinetics, is also an important consideration in assessing cytotoxicity. Many studies have examined the *in vivo* distribution of nanomaterials [26]. Nanoparticles with a diameter greater than 6 nm cannot be excreted by the kidneys and accumulate in specific organs, such as the liver and spleen, until clearance by the mononuclear phagocyte system ensues [27]. Most nanoparticles that accumulate the in liver and spleen cause serious side effects. For instance, cadmium selenide (CdSe) quantum dots remain in the tissue for up to eight months and cause hepatotoxicity [28]. This pharmacokinetic characteristic of nanoparticles is dependent on particle size and surface chemistry.

**Figure 3:**
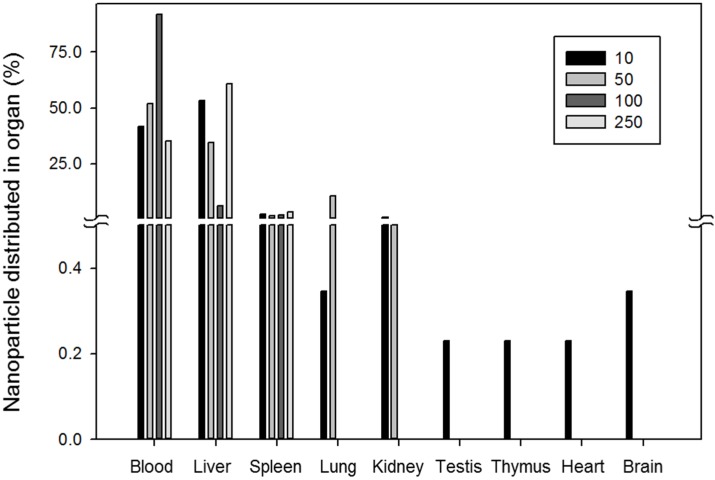
Gold nanoparticle distribution in several organs in rat according to particle size (nm).

The *in vivo* distribution of gold nanoparticles according to size was evaluated by De Jong *et al*. [29]. They used particles from 10 to 250 nm in size and assessed *in vivo* distribution after intravenous injection in a rat model. They found that 10 nm nanoparticles were distributed differently than their larger counterparts. They were found in almost all organs, including the blood, liver, spleen, kidneys, testes, thymus, heart, lungs and brain. Meanwhile, most nanoparticles larger than 50 nm were detected only in the blood, liver and spleen. The distribution of nanoparticle in several organs is shown in Figure 3. This figure is reproduced from the work of De Jong *et al*. [29].

### 2.3. Size-Dependent Cellular Uptake and Cytotoxicity

In terms of cellular interaction, nanoparticle uptake mechanism and efficiency are key factors influencing cytotoxicity. One of the major factors determining cellular uptake efficiency and mechanism is nanoparticle size. With respect to particle size and surface features, nanoparticles are internalized into the cell through various pathways, such as phagocytosis and pinocytosis. Several internalization pathways depend on size as noted in Table 2 [30,31]. Sizes suitable for uptake range from 10 to 500 nm with an upper limit of 5 mm. Large particles are most likely to be engulfed via macropinocytosis. The size of a vesicle involved in clathrin-mediated endocytosis is about 100 nm, while the size involved in caveolae-mediated endocytosis is usually about 60–80 nm. However, the examples in the literature also cite the uptake of particles larger than those size ranges.

**Table 2:** Examples of cellular internalization pathways of nanoparticles.

Internalization pathway	Materials of particle	Particle diameter (nm)	Ref.
Clathrin-mediated endocytosis	[C_60_(C(COOH)_2_)_2_]*_n_*	125	[32]
	PVA coated silver NP	80	[33]
	PEGylated NP	90	[34]
	LDH NP	50–200	[35]
	QD	4	[36]
	Polystyrene NP	100	[37]
	Pristine PS NP	50–200	[38]
	Silica NP	110	[39]
	Herceptin–collidal gold NP	2–100	[40]
	Silica coated iron oxide NP	20	[41]
	AuNR		[42]
	bombesin peptide conjugated AuNC		[43]
Caveolae-dependent endocytosis	Derivatized fullerenes Baa-Lys(FITC)-(Lys) 8-OH	4	[44]
	Perfl uorocarbon NP	200	[45]
	Polysiloxane NP	100	[46]
	fWGA–PLGA NP	250	[47]
	Albumin-coated NP	20–100	[48]
	AuNR	56 × 13	[49]
Pinocytosis/Macropinocytosis	PVP-coated silver NP	80	[33]
	Positively charged fluorescent polystyrene NP	113	[50]
	Tat peptide-conjugated QD		[51]
	Silica NR		[52]
	Silver NP	25	[53]
	IL-13 peptide conjugated PEG-PCL NP	25–100	[54]

Gold nanoparticles typically form a surface coated with serum proteins when incubated with cells. Serum-layered gold nanoparticles usually induce receptor-mediated endocytosis, which is dependent on particle size. The uptake efficiency of gold nanoparticles as a function of size was evaluated by Chan *et al*. [55]. Gold nanoparticles ranging from 1 to 100 nm were incubated with Hela cells, and the 50 nm nanoparticle showed maximal uptake efficiency by receptor-mediated endocytosis [55]. A similar experiment using ligand-coated gold nanoparticles showed that a diameter of 40–50 nm was the critical cutoff point for receptor-mediated nanoparticle internalization [40]. This phenomenon is tightly related to the nanoparticle’s binding and its cellular surface receptors. Extremely small nanoparticles have a surface curvature too great to provide necessary conformational rigidity to allow for multivalent binding with receptors. In contrast, larger nanoparticles cannot compensate for the depletion of receptors within the binding area through global diffusive motion of distant receptors, and this could limit the process of membrane wrapping that is necessary for nanoparticle internalization. As such, nanoparticles that are 40–50 nm in diameter seem to be the optimal size for both multivalent receptor interaction and binding rigidity.

Differences in endocytosis efficiency naturally affect cellular cytotoxicity. The internalized nanoparticles generally translocate to endosomal or lysosomal vesicles for further elimination [16]. During this process, endosomal escape of internalized nanoparticles occurs, resulting in specific cytotoxicity through the production of ROS and direct mitochondrial damage.

## 3. Surface

In addition to size and surface charge, the particle surface itself also plays a critical role in biological toxicity.

### 3.1. Surface Area

Band gap alterations, decreased melting point, and higher reactivity induced by a large surface area were investigated [56,57,58] and it was found that these features had serious effects including lung inflammation, cytotoxicity and toxicity *in vivo*. A larger surface area may cause higher reactivity with nearby particles, resulting in possibly harmful effects when used in fillers, cosmetics, and as drug carriers [59]. It can be concluded that by decreasing the particle size, its biological activity increases substantially. Smaller particles occupy less volume, such that a larger number of particles can occupy a unit area, resulting in increased pathophysiological toxicity mechanisms, for instance oxidative stress, ROS generation, mitochondrial perturbation, *etc*. [60]. It has yet to be determined what features of nanoparticles cause such biological toxicity. It is presumed that the size of the nanoparticle alone may not be responsible for toxicity, but that the total number per unit volume may be important. To clearly comprehend the relationship between a nanoparticle’s surface area and its biological toxicity, a group of researchers assessed acute lung inflammation with different nanoparticle surface areas and specific reactivity [61]. There were no significant differences in toxicity differences based on size; however, the total surface area played a critical role in lung inflammation. It has been clearly confirmed that pulmonary toxicity, assessed after treatment with several particles including carbon black, titanium dioxide, *etc.*, may be induced by ultrafine and fine materials [62,63,64], which have large surface areas. Particle surface reactivity, characterized by how easily single particles aggregate, may also play a significant role in cytotoxicity [65,66,67,68]. It has also been suggested that dosing should be based on nanoparticle surface area.

### 3.2. Surface Electrostatic Status

Due to their small size, nanoparticles are usually used as a drug carrier via either passive or active transport. Their effective cellular internationalization depends upon biocompatibility [27,69,70]. In particular, external properties of surface electronic status are critical to cellular uptake and may also be involved in cytotoxicity. Traditionally, to study *in vitro* efficacy, nanocarriers are instilled into a 2D layered target cell for both therapeutic and diagnostic studies. However, such methodology should be reconsidered prior to *in vivo* study, because such a layered model may be dissimilar to that of a cell niche where cell to cell communications are critical for metabolic progress. Nonetheless, nanoparticles for delivery are currently designed based on *ex vivo* conditions mimicking tissue environments [71,72]. Similar methods are used to assess nanoparticle toxicology [73,74]. Higher uptake efficiency in a cell is achieved by replacing the surface functional moiety, inducing sudden changes in particles’ surface charge [75,76]. Some authors have attempted to envelope nanoparticles in a lipid vesicle [77].

Changes in surface charge result in considerable differences in the *in vivo* biodistribution of nanoparticles. Even though some nanoparticles, including polymeric nanocomplexes and gold nanospheres, have the same range of size variations, different surface charges yielded marked discrepancies in both distribution and uptake efficiency. Particles showed different degrees of toxicity depending on their surface charges [78,79,80,81]. Nanoparticles with a positively charged surface tended to have much higher toxicity.

## 4. Morphology

Morphology is also a big issue in nanotoxicology. Like other well-established inhalable fibers (e.g., asbestos), nanoscaled fibers (e.g., carbon nanotubes) are reported to have a serious risk of lung inflammation. Furthermore, prolonged exposure may cause several cancers [82,83,84]. It is difficult to determine whether there is a certain toxic effect of single nanotubes or an ensemble of such tubes. Some studies have shown that carbon nanotubes are more toxic than other ultra-fine carbon black or silica dusts. Most workers exposed to single-walled carbon nanotubes (SWCNTs) beyond the current permissible exposure limit (PEL) developed lung lesions [85]. Interestingly, CNTs have been shown to cause death of targeted kidney cells via inhibited cell growth induced by decreased cell adhesiveness [86]. Human exposure to fullerene (also termed Buckyball) resulted in severe lung damage [87,88] in addition to destruction of fish brain and water flea death [89,90,91,92].

## 5. Agglomeration Status

Irrespective of any physicochemical properties of nanoparticles, such as chemical composition, agglomeration could be a potent inducer of inflammatory lung injury in humans [93,94]. For certain types of chemicals, exposure at higher levels has been shown to lead to serious chronic diseases such as fibrosis and cancer [95]. It is still under consideration to figure out what features are inducing such toxicological effect in a living organism.

## 6. Summary

The ability to engineer nanometer-size materials has been proven to have great value in several fields, including basic science, such as chemistry, physics, biology and even engineering fields like biotechnology and electronics. Prior to the widespread introduction of such nanotechnology in medicine, its safety in biological systems should be investigated thoroughly. All physicochemical properties of nanoparticles should be evaluated in order to elucidate their interaction with subcellular organelles, cells, tissues, and organisms. Such investigations will provide us with strategies to engineer new generations of nontoxic products containing nanoparticles. These fundamental studies will help to generate criteria for the smart design of nanoparticle which can be used *in vivo*.
